# Effect sizes in human functional neuroimaging

**DOI:** 10.21203/rs.3.rs-8896956/v1

**Published:** 2026-06-05

**Authors:** Stephanie Noble, Hallee Shearer, Matt Rosenblatt, Jean Ye, Rongtao Jiang, Link Tejavibulya, Maya Foster, Qinghao Liang, Javid Dadashkarimi, Margaret Westwater, Iris Cheng, Max Rolison, Hannah Peterson, Brendan D. Adkinson, Saloni Mehta, Chris Camp, Alexandra Fischbach, Fabricio Cravo, Amanda Mejia, Thomas Nichols, Joshua Curtiss, Dustin Scheinost

**Affiliations:** 1. Department of Psychology, Northeastern University; 2. Department of Bioengineering, Northeastern University; 3. Institute for Cognitive & Brain Health, Northeastern University; 4. Center for Precision Psychiatry, Department of Psychiatry, Massachusetts General Hospital; 5. Psychiatric and Neurodevelopmental Genetics Unit, Center for Genomic Medicine, Massachusetts General Hospital; 6. Psychiatry, Massachusetts General Hospital; 7. Department of Biomedical Engineering, Yale University; 8. Interdepartmental Neuroscience Program, Yale University; 9. State Key Laboratory of Cognitive Neuroscience and Learning, Beijing Normal University, Beijing, China; 10. Perelman School of Medicine, University of Pennsylvania; 11. Department of Radiology & Biomedical Imaging, Yale University; 12. Child Study Center, Yale School of Medicine; 13. Department of Psychiatry and Behavioral Sciences, Boston Children's Hospital; 14. Department of Statistics, Indiana University; 15. Big Data Institute, Li Ka Shing Centre for Health Information and Discovery,Nuffield Department of Population Health, University of Oxford; 16. Oxford Centre for Integrative Neuroimaging, FMRIB, Nuffield Department of Clinical Neurosciences, University of Oxford; 17. Department of Applied Psychology, Northeastern University

## Abstract

Emerging reports suggest that sample sizes commonly used in functional neuroimaging studies may be too small to detect many brain-behavior relationships, posing a major barrier to brain and mental health research. A central challenge is that planning robust studies requires researchers to know what effect sizes to expect, yet this essential information is surprisingly difficult to estimate in practice and thus often omitted from study planning. Critically, standard “mass univariate” procedures for estimating effects across multiple brain areas give an inflated picture of how large effect sizes are. Here, we introduce a method to correct this inflation bias and perform an unprecedented analysis of 63 studies in seven large datasets (n = 100–40,000; 52,979 total participants) to establish effect size benchmarks in functional neuroimaging. We find that between-subjects effects are exceedingly small at the majority of brain areas (Cohen’s ∣*d*∣ < 0.2), requiring consortium-level sample sizes to detect even some of the strongest focal brain effects (n > 500 at 80% statistical power with FDR correction). However, multivariate analyses and within-subject task designs yield substantially larger effect sizes that can be detected at sample sizes within reach of individual labs (n < 50). By establishing data-driven effect size benchmarks, these findings lay the groundwork for more informed study planning in neuroscience while highlighting shared challenges (and the potential for shared solutions) across biomedicine.

## Introduction

Human neuroimaging research has blossomed over the past decades, spurred in part by major international efforts to understand the brain such as the US BRAIN Initiative and the International Brain Initiative. These efforts have transformed our understanding of brain function in health and in disease. However, as the field has matured, mounting evidence has revealed critical issues with the replicability of many findings, including in the domain of functional magnetic resonance imaging (fMRI; e.g.^123^; see also “reproducibility crisis”). These critiques often highlight the limited availability of information needed to guide the design of more robust and reliable studies. Central to these challenges is a deceptively simple yet fundamental question: what effect size should I expect when planning my study?

This question seems straightforward enough to answer. After all, the most common study planning tool researchers possess—the power analysis—requires knowing the expected effect size in order to calculate how many subjects are required to detect it. Given the fundamental reliance on effect size estimation, one might expect it to be well characterized. However, answering this question is not as easy as it seems. While anecdotal reports suggest that effects are small, little concrete evidence exists to quantify the exact size of effects. Effect sizes in neuroimaging are influenced by numerous factors, including the type of effect (e.g., univariate versus multivariate), study design (e.g., within-subject versus between-subject), outcome measure under investigation (e.g., cognitive versus biometric measures), brain imaging modality (e.g., task-based activation versus functional connectivity), and more. While some effects have been reliably replicated—such as the presence of many large scale networks^4^—others, such as common “brain-behavior associations” (i.e., relationships between functional connectivity and behavior), are less reliable and suggested to be small (e.g., ^2^). Adding to this complexity, despite increasing statistical guidance to supplement p-value reporting with effect sizes^5,6^, effect size reporting in neuroimaging remains inconsistent. It often focuses on the location of maximally significant effects, less frequently includes actual effect size magnitudes, and, due to complications involving the high dimensionality of the data, rarely provides estimates of uncertainty that are critical for accurately interpreting effect sizes (for one example of the increasing emphasis on uncertainty reporting, see ^7^).

Indeed, the issue of sampling variability complicates this problem. Effect size “point estimates” are only approximations of the true population effect, and their accuracy declines with smaller sample sizes. Historically, neuroimaging studies have relied on small samples—often around 25 participants^8^—resulting in effect size estimates with errors that could vastly exceed the actual magnitudes. Researchers may then base their study designs on these uncertain estimates or rely on pilot studies with even smaller sample sizes, resulting in even greater uncertainty. To circumvent these challenges, researchers often default to using sample sizes observed in prior “successful” studies, implicitly assuming that those effects are representative of their own study. Whether implicitly or explicitly adopting the effect sizes of “successful” studies, relying on prior point estimates without accounting for uncertainty can be misleading because these estimates are often inflated due to selective reporting practices (cf. "winner’s curse" and "file drawer problem”). This leads to a feedback loop that reinforces inflated expectations and makes it even harder to accurately estimate effect sizes in neuroimaging.

Efforts to address issues with reproducibility and replicability, such as those discussed above, have gained momentum in recent years, yet these efforts have typically focused on null hypothesis testing (i.e., whether a finding is significant rather than its magnitude) or touch on effect size in indirect or limited ways (e.g., for isolated studies). Moreover, we have not yet witnessed a systematic effort to estimate the distribution of effect sizes across the brain while accounting for the uncertainty that arises from examining thousands of brain variables simultaneously, which can produce inflated estimates of effect size magnitudes when effects are small and errors large (cf. Methods*: Estimating population effect size distribution*). A systematic effort is needed to provide more robust effect size benchmarks to inform study planning in functional neuroimaging. Only recently, with the proliferation of large, publicly shared neuroimaging datasets alongside advances in computational infrastructure to analyze them, has such a comprehensive investigation become possible.

The present work leverages multiple large-scale neuroimaging datasets to provide a systematic estimation of effect size distributions across typical functional brain imaging studies. To our knowledge, this represents the first comprehensive effort to estimate the “true” distribution of effect sizes across the brain for the common fMRI study designs, establishing empirical foundations for more robust evidence-based study planning in the field. This effort not only supports the increased movement towards transparent reporting in human brain mapping, but also connects the field with statistical developments in other biomedical fields facing similar challenges in high-dimensional data (e.g., ^9^).

### Effect sizes in functional neuroimaging

**Figure 1.**
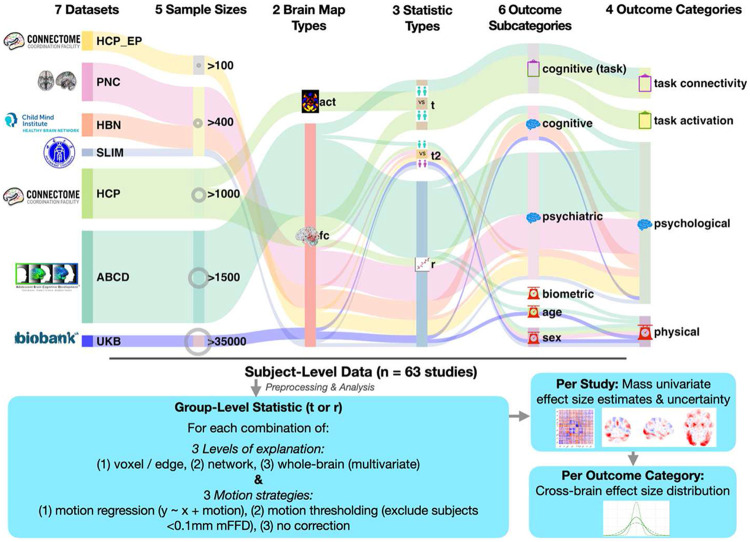
Study characteristics and effect size estimation workflow. Top, sankey diagram depicts datasets, sample sizes, brain map types, effect test types, outcome sub-categories, and outcome categories for each study in the database, with width of each segment of the pathway representing the number of studies in that segment. Bottom flowchart depicts the process of converting subject-level statistical maps to group-level statistical maps and then effect size measures. See **SI Tables 1-2** for detailed study characteristics.

**Figure 2.**
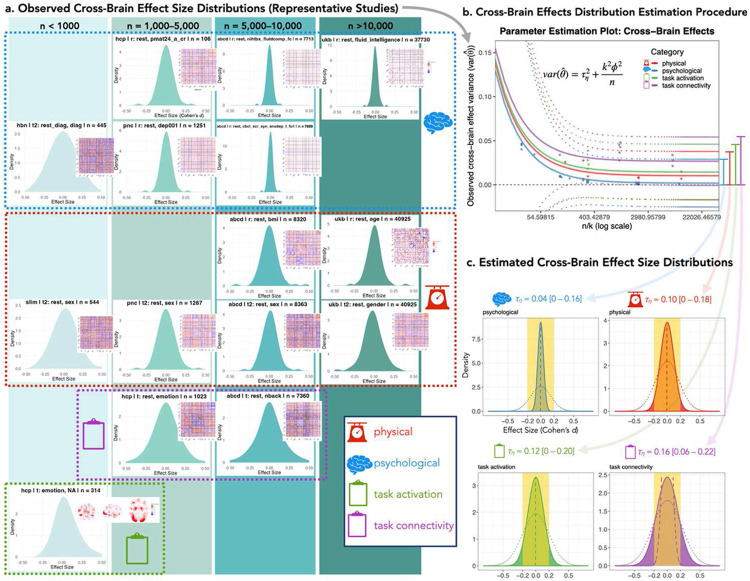
Estimating the corrected distribution of effect sizes across the brain reveals consistently small magnitude effects. **(a)** Mass univariate effect size distributions for exemplar studies. Studies are arranged by sample size (left to right; also light-to-dark density plot colors) and outcome category (top to bottom boxes). **(b)** Procedure for estimating the distribution of effect sizes across the brain for each outcome category. Parameter estimation plots illustrate how the per-study mass univariate cross-brain variance $var(\hat{\theta })$ decreases with sample size n, asymptotically approaching the true value $\tau_{n}^{2}$ at a rate that depends on the number of test groups k (e.g., k=1 for 1-sample tests) and within-variable sampling error $\phi^{2}$. Points indicate $var(\hat{\theta })$ for each study. **(c)** Estimated effect size distributions for each outcome category: psychological (blue), physical (red), task-based activation (green), and task-based connectivity (purple). Density plots are shown for parameter point estimates (solid line) and 95% confidence interval upper and lower bounds (dotted lines). Gold bars indicate effects below the conventional “small” (*∣d∣*<0.2) effect size threshold. Estimated distributions reveal that the majority of univariate neuroimaging effect sizes fall well below conventional effect size benchmarks. Abbreviations: Adolescent Brain and Cognitive Development: ABCD; confidence interval: CI; Healthy Brain Network: HBN; Human Connectome Project; HCP; Philadelphia Neurodevelopmental Cohort; PNC; UK Biobank: UKB.

We performed 63 studies spanning psychological (i.e., cognitive, psychiatric), physical (e.g., age, sex/gender, body mass index; see caveats in Methods), and task-based research across seven datasets (total n = 52,979 unique participants) representing diverse populations, study designs, and outcome types (Fig. 1; detailed study characteristics in **SI Tables 1-2**). When performing a study, researchers typically estimate “mass univariate” effect sizes for each brain area separately and report the full map of point estimates across the brain (e.g., exemplar studies in Fig. 2a; all studies in **SI Figs. 1-2**). However, these mass univariate distributions are inflated—specifically, they exhibit higher variance than the true distribution due to sampling error, narrowing and approaching the true width as sample size increases (cf. Fig. 2a). Following in the spirit of a meta-analysis, we developed a principled approach that uses multiple studies to model this decreasing variance and estimate, for the first time, the true, uninflated distribution of effect sizes across the brain (Fig. 2b; Methods*: Estimating population effect size distribution*).

### Edge- and voxel-level effects are generally small in neuroimaging studies

Across study categories, we found that the majority of effects (79% to >99%) fall below the *∣d∣* = 0.2 threshold conventionally considered “small” (^14^; Fig. 2c; **SI Tables 3-4**). Between-subjects studies, such as brain-behavior associations or group contrasts, exhibited particularly small effects: for a typical study examining physical and psychological variables, 95% and >99% of effects respectively exhibited effects below *∣d∣* = 0.2, with only 1% and 32% surpassing the even smaller *∣d∣* = 0.1 mark.

Motion correction strategies that do not directly regress out motion effects (no correction or excluding subjects with >0.1mm mean frame-to-frame displacement) resulted in larger effect sizes than motion regression, with most between-subject effects remaining small (∣d∣ < 0.2) but many task-based effects approaching a medium effect size (∣d∣ < 0.5; **SI Fig. 3**). The fact that these conditions yield such similar results points towards the presence of substantial motion-related effects that persist even when excluding high motion subjects.

By estimating effect size distributions while correcting for magnitude inflation, these findings confirm that effect sizes are not just small, but substantially smaller across study types than existing isolated and uncorrected reports suggest (e.g., ^2,3^).

Researchers designing similar neuroimaging studies would benefit from anticipating small effects and planning accordingly.

#### Task connectivity and physical studies exhibit larger effects

While between-subject effects of all types were generally small, physical studies exhibited effect sizes that were approximately six times larger than those of psychological studies. In contrast, task-based (within-subject) connectivity effect sizes were more than double those found in physical studies (Fig. 2b). In fact, nearly a quarter of task-based connectivity effects exceeded the *∣d∣* = 0.2 threshold conventionally denoting small effects. Note that task-based studies rely on within-subject designs, whereas the physical and psychological studies used here represent between-subject designs. The larger effect sizes for within-subject studies are partially expected due to the reduced impact of substantial between-subject error.

However, even for the same tasks and dataset, task-based activation effects were about half the size of task-based connectivity effects. Some task-based activation effects may even be potentially on par with some physical brain-wide association effects. This may reflect a number of intrinsic methodological differences—such as connectivity analyses using distinct rest scans as a contrast while activation analyses compare states within a single scan, in addition to the much smaller resolution of voxels for activation compared with regions for connectivity—but warrants further investigation. Altogether, the present results suggest that researchers should plan for most psychological associations to be very small (explaining the low replicability of cognitive and psychiatric associations previously reported by ^2^), physical associations to be moderately larger but still small, and task-based effects to be small to medium.

### A closer look at variability across study types

**Figure 3.**
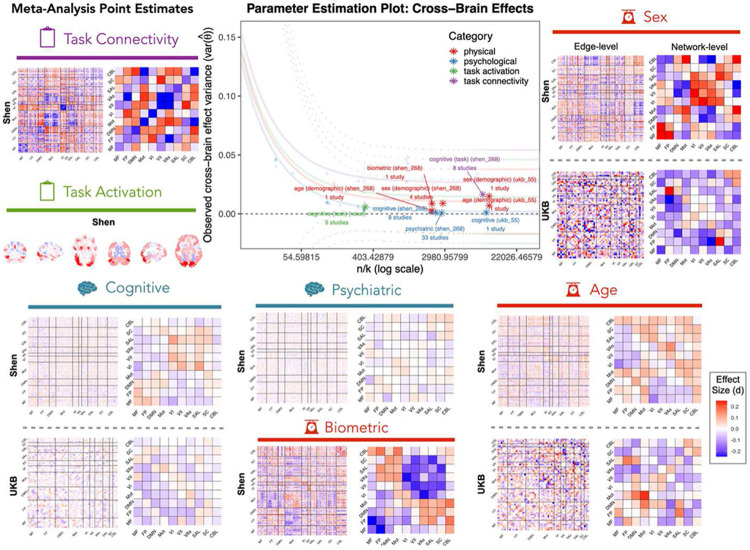
Additional heterogeneity across outcome sub-categories. Results shown for each subtype within each outcome category (red, physical; blue, psychological; green, task activation; purple, task connectivity). Meta-analysis results are depicted for sub-categories containing more than one study and do not include UKB studies due to the unique parcellation used to obtain edge-level results. For UKB and sub-categories containing a single study, study-level results are shown. **Middle panel:** $\tau_{n}^{2}$ estimation plot (transparent) overlaid with $var(\hat{\theta })$ from meta-analysis results for each sub-category (stars). **Surrounding panels:** Spatial plots of effect size point estimates (uncorrected) for each category, with variables arranged by canonical networks (functional connectivity maps) or spatial location (activation maps). Under each category is shown edge- or voxel-level results (left) alongside network-level results (right), and for Shen (top) and UKB (bottom) atlases as available. Brighter red indicates larger positive effects; brighter blue indicates larger negative effects.

Subtypes within outcome categories were further explored, using meta-analyses to extract prevailing effects within subtypes featuring more than one study. The relative positions of outcome sub-categories on the parameter estimation plot (Fig. 3) suggests that sex differences may potentially surpass age-related effects. However, many pooled effects (e.g., task-based connectivity) showed smaller magnitudes than may be expected from the individual study-level data, potentially revealing limitations regarding which studies can be meaningfully combined. Note that pooling specifically emphasizes shared effects across studies and attenuates effects that are unique to any particular study.

#### Spatial distribution of effects

The spatial distribution of pooled effects varied by category and dataset, often featuring results that clustered within functionally related networks including task-related areas (medial frontal, frontoparietal, default mode) and visual areas (VI, VII, VAs; Fig. 3). These functional clusters showed distinct patterns across categories: cognitive scores showed positive associations with connectivity within these clusters, whereas body mass index showed negative associations, and task states showed reduced connectivity in these clusters. These clusters showed more positive connections in women than men, although UKB showed more negative effects than ABCD that may be attributed to either differences in preprocessing or age in those datasets. Indeed, age associations differed substantially across datasets, though all showed modestly increased connectivity within task-relevant networks with the exception of medial frontal-medial frontal connections. Psychiatric effects were relatively diffuse across brain regions.

The default mode network (DMN) showed notable deviations from the clustering within functionally related areas described above. During task states, while most connections within the task-related cluster decreased, DMN connections to other task networks actually increased (note that this is visible at the network-rather than edge-level). Similarly, whereas body mass index was negatively associated with most cluster connections, only within DMN associations increased.

Pooled task-based activation maps displayed areas of relative intensity across the map, including extensive activations across occipital and frontoparietal areas and deactivations along various sensory and motor areas. Looking more closely, functional connectivity was more similar across specific tasks whereas activation maps differed more substantially (**SI Fig. 2; SI Fig. 4**), potentially reflecting both the more precise modeling of task-specific phenomena during activation analysis and the dominant influence of resting-state in the connectivity contrast.

### Larger effects at broader and multivariate scales

**Figure 4.**
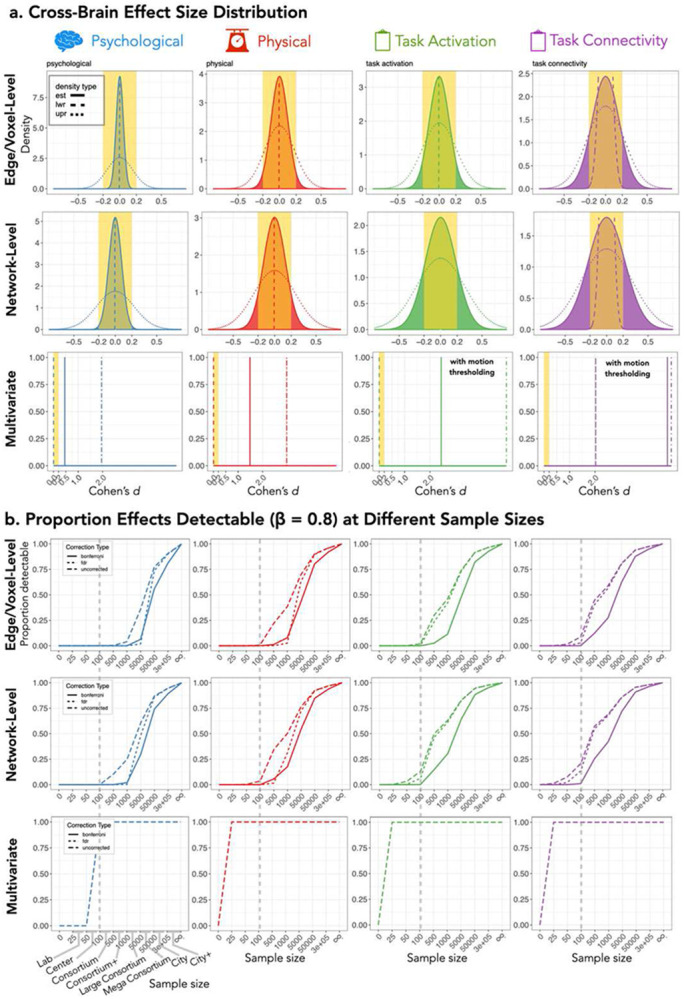
Statistical power analysis reveals the need for larger sample sizes and broader-scale analyses to detect effects. **a)** Edge- or voxel-level (top), network-level (middle), and multivariate (bottom) estimates of effect size distributions for each outcome category (red, physical; blue, psychological; green, task-based). For multivariate within-subject results only, motion thresholding rather than motion regression was used since the motion regression procedure does not exist for a one-sample multivariate t-test. Gold bars indicate effects below the conventional “small” (*∣d∣*<0.2) effect size threshold. **b)** Sample size required to detect effects with 80% power for edge- or voxel-level (top), network-level (middle), or multivariate (bottom) effects for each outcome category (red and top, physical; blue and middle, psychological; green and bottom, task-based). Each plot shows the proportion of effects across the brain that can be detected with 80% power in each sample size category after Bonferroni, false discovery rate (FDR), or no correction. Sample size categories, chosen to align with observed practices in the field, include: Lab (n = 0–24), Lab+ (n = 25–49), Center (n = 50–99), Consortium (n = 100–499; e.g., HCP-EP; SLIM), Consortium+ (n = 500–999; e.g., PNC, HBN), Large Consortium (n = 1,000–4,999; e.g., HCP), Mega Consortium (n = 5,000–49,999; e.g., UKB), City (n = 50,000–299,999), and City+ (n = 300,000+). A grey dotted line denotes sample sizes expected to be attainable at individual sites versus those requiring multiple sites (i.e., Consortium).

Network-level (i.e., pooled within network) and multivariate (i.e., CCA and Hotelling’s T2 test) effects were modestly (network) to substantially (multivariate) stronger than edge- or voxel-level effects (Fig. 4a; **SI Tables 3-4**; **SI Fig. 5**). This explains, in part, the greater power and replicability found with large scale and multivariate methods (^2,2,3,1011^). However, it should be noted for multivariate effects that studies can produce the same overall effect size magnitude without necessarily producing the same actual vectors (for example, opposite signed vectors can produce the same magnitude).

### Study planning implications from traditional power analysis

To illustrate the practical implications of the effect size results, we performed a mass univariate parametric power analysis (Fig. 4b), which mirrors power analyses conventionally used for study planning in psychology (e.g., ^12,13^). To accomplish this, we developed a framework to estimate mass univariate power given a cross-brain distribution of effect sizes under different multiple testing control procedures including Bonferroni, false discovery rate (FDR), and no control settings (see *SI Methods: Estimating cross-distribution power with multiple testing correction*).

As expected by the prevalence of very small effects, the vast majority of univariate effects could not be detected with adequate power (β = 80%) at Consortium-scale sample sizes (n=100 subjects). Between-subjects studies required Large or Mega Consortium-scale sample sizes to detect more than half of all brain effects, even without multiple comparison correction (n > 16,000 for psychological; n > 2,000 for physical). Task-based effects were more detectable; detecting half of all effects required approximately half the sample size for task-based connectivity compared with physical outcomes (e.g., n ≈ 1,000 vs. n ≈ 2,000 uncorrected; n ≈ 2,000 vs. n ≈ 4,000 with FDR; n ≈ 5,000 vs. n ≈ 13,000 with Bonferroni), and substantially fewer subjects for task-based activation (e.g., n ≈ 700 uncorrected; n ≈ 800 with FDR; n ≈ 3,500 with Bonferroni). Some task activation effects were even detectable at sample sizes attainable within individual sites (n < 100), partially reflecting the power advantages of within-subject designs even at the same magnitude effect size as between-subject designs (**SI Table 5**).

Power likewise increased substantially with broader-scale approaches. At the network level, a non-negligible proportion of task-based effects were detectable at the Center level with FDR correction (~6%–12%). More than a quarter of physical effects became detectable at the Consortium+ scale with FDR correction, though psychological effects still required Large Consortium sample sizes. Critically, all multivariate effects were detectable at sample sizes within reach of individual labs^8^ for physical and task-based studies (n ≤ 25) or individual sites for psychological studies (n ≤ 100).

Power also varied as expected across multiple testing approaches (i.e., uncorrected > FDR > Bonferroni). Yet, notably, power with FDR correction approached that without correction for task studies above n = 100, underscoring its practical utility. Power also varied as expected across motion deconfounding strategies, with no deconfounding and thresholding yielding higher power than regression (**SI Fig. 6–7**), again likely reflecting unmitigated motion-related effects.

Overall, traditional power analysis suggests that detecting univariate between-subject requires consortium-scale samples, supporting the suggestion by ^2^ that large-scale collaborative efforts may be necessary for replicable brain-behavior association research. However, broader-scale and multivariate approaches substantially reduce these requirements, enabling adequately powered studies at sample sizes within individual labs or sites.

### Turning anecdote into evidence for study planning

In the functional neuroimaging community, it is a common refrain that effect sizes are small and sample sizes inadequate (cf. ^2^). Although these concerns have been supported by anecdotal accounts and isolated findings, there has not yet been a comprehensive investigation into these claims. Any such investigation must also contend with the uncertainty that arises when examining thousands of variables simultaneously. In this study, we examined a variety of commonly used study types in some of the largest datasets currently available to directly assess the magnitude of effect sizes in fMRI, developing a method to correct for cross-brain effect size magnitude inflation. Our findings align with the prevailing expectation that fMRI effects are small but further demonstrate that they are much smaller than commonly suggested, such that typical labs are often underpowered to detect them. There is a clear need to revisit common study design and analysis practices so that researchers are better positioned to detect subtle effects.

Although revision is warranted, the situation is not as dire as it seems. Brain imaging is a relatively new and impressive field, and, in the prescient words of Cohen, “In new areas of research inquiry, effect sizes are likely to be small (when they are not zero!) [because they are] typically not under good experimental or measurement control or both”^14^. Indeed, best practices for measurement, acquisition, analysis, and more are continuously being refined for neuroimaging in the face of its complex and historically unwieldy data characteristics, and patience is likewise deserved when studying such obscure, individualized phenomena as mental life^15^. The scale of neuroimaging discoveries that are replicable remains impressive (e.g., the ability to map critical language cortex during surgery, the discovery of classes of large scale brain networks, etc.), especially keeping in mind that even relatively well-characterized phenomena exhibit “small” effect sizes (e.g., the difference in IQ similarity between twins and non-twins; the height difference between 15 and 16 year old girls^14^).

#### Planning conservatively for future studies

The results presented in this study provide a concrete touchstone for researchers to reflect on how they approach planning future fMRI studies. While our findings largely support the expectation of small univariate effect sizes for most study types, we also highlight that the situation is not monolithic. For example, within-subject task-based designs can increase effect sizes to the extent that such researchers would be able to design studies that are fundamentally different in scope and scale. If analyses focus on highly specific task-based tests, fewer than 25 subjects can be used to detect some of the strongest effects.

Our results also emphasize that larger-scale inference across brain regions can meaningfully increase effect sizes and power, consistent with previous findings^3^. We demonstrated this power benefit even in the absence of multiple comparison correction; the need to correct for fewer numbers of tests over large-scale compared with edge- and voxel-level approaches is expected to further accentuate this difference. Multivariate approaches showed a particular benefit and are further known to be more reliable and replicable ^2,10,11,16^. Altogether, this work adds to an increasing body of literature that supports the idea that combining information across the brain captures robust, widespread effects that are often overlooked when focusing solely on spatially localized signals (cf. ^15^).

Our approach to estimating effect size distributions was derived from basic theoretical principles regarding sampling error that we extended to empirically characterize effect size distributions in high-dimensional neuroimaging data. This strategy avoids the limitations of using either marginal estimates from traditional simultaneous confidence intervals (too uncertain given the high dimensionality) or point estimates from individual studies (inflated magnitude due to sampling variability; cf. *Supplementary Methods: Estimating population effect size distribution*). To our knowledge, these distributions provide the first empirical estimate of benchmarks for study planning in neuroimaging. In the absence of other empirical benchmarks, we recommend researchers consider both the main distribution parameters and their uncertainty when planning studies, while bearing in mind the below limitations in generalizability and precision of these estimates.

Overall, these findings invite researchers to rethink their reliance on traditional study planning methods and embrace empirically informed methods that incorporate uncertainty^17,18^. In particular, researchers should exercise caution when basing power analyses on point estimates from individual studies, as the potential for both magnitude and sign errors when effects are small relative to sampling error makes such calculations particularly questionable. To facilitate a deeper understanding of study level estimates and their uncertainty, we previously released an interactive Shiny app^19^ that enables researchers to explore all uncorrected mass univariate effect size estimates used in the present study (e.g., **SI Fig. 2, 4**). Note that while uncorrected estimates have many uses, they should not directly be used for study planning given the inflation bias discussed in the present work.

#### Limitations

This study represents a first step toward creating a more comprehensive database of standard effect sizes for a wide range of fMRI study types. As such, we aimed to start by including some of the most common and simple study design and analytical procedures we could find, using some of the largest datasets currently available, and summarize this information by estimating distribution parameters within study categories. However, several critical extensions would greatly broaden the scope and utility of this resource.

The current findings have limited generalizability beyond the study categories included here and would benefit from inclusion of additional task-based studies, within-subject contrasts, longitudinal processes, scan protocol variations (including scan length; cf ^20^), and other processing and analysis decisions known to substantially impact results^1,7^, particularly timeseries-level artifact attenuation strategies. We also grouped studies into categories based on outcome variables for simplicity, but there are of course important differences between studies within a category (e.g., age differences in UK Biobank compared with other datasets) that would benefit from future investigation.

Expanding the database to incorporate smaller-scale studies, such as those hosted on platforms like NeuroVault^21^, would also be an important next step. Smaller studies are abundant and diverse, representing a wealth of untapped information from a broader range of research contexts than is captured by the select few large datasets included here. However, integrating these studies presents unique challenges around data standardization to facilitate aggregation. We introduced a new contribution format to help navigate this^19^, but it will also be important to explore how this new format can align with existing standards (e.g., ^22^) and facilitate manual labeling of categories for aggregation which could quickly become impractical with the large number of smaller-scale studies. It is also worth considering how one might integrate results from publication-based meta-analyses (e.g., BrainMap^23^ and NeuroSynth^24^), which represents another avenue for capturing the wealth of existing results to inform effect size estimates.

Finally, we used an approach to estimate population distribution parameters by modeling how effect size variability changes with sample size asymptotically, but this method has inherent limitations and lacks complete generalizability across all neuroimaging contexts. Our distributional approach provides realistic benchmarks by correcting for magnitude inflation, but additional work is needed to characterize and improve the precision of these estimates across diverse study types and populations. An important note is that we have quantified univariate, network-level, and multivariate effects and power, but have not comprehensively estimated power for common fMRI-specific inferential techniques (e.g., cluster-based inference; cf. ^25^ for power estimation in HCP task activation data). To support future methodological developments, we have made the full set of effect sizes and estimation procedures publicly available.

#### Future Directions

It is not only essential to characterize effect sizes for the sake of informing future neuroimaging research, but also to contextualize findings and share solutions from other medical disciplines. For example, genetic studies are known to exhibit similarly small effects across often millions of common genetic loci, requiring hundreds of thousands to even millions of subjects for detection^26^. The field of statistical genetics has spent more than a decade examining how to best estimate effect size distribution parameters in high-dimensional data while accounting for widespread, correlated effects^9^. While it is unclear to what extent genomic assumptions may extend to neuroimaging data (e.g., linkage disequilibrium, the expectation of sparse causal factors), the field would benefit from greater reflection on advances in statistical genetics and how they may be applied to neuroimaging (cf. ^26,27^). In contrast, behavioral psychology effect sizes can be an order of magnitude larger, although reports are often inflated^28,29^. A useful idea from psychology is to revise our expectations and better appreciate the implications of likely small effects (cf. ^18,29^). Future work aimed at incorporating findings and advances from other fields will help us better understand the pragmatic implications of brain-based effects.

Beyond informing traditional study planning, this work is intended to support methodological advances in neuroimaging. Efforts are underway to develop a power calculator that relies on empirical effect size estimates^30^, and we anticipate the development of additional tools that leverage the structure and magnitude of estimated effects to inform or constrain estimation of effects in individual studies. To facilitate this process, we provide an interactive Shiny app that allows users to explore effect size distributions and tailor their planning to the specific characteristics of their intended study. The long-term success of this and a burgeoning variety of related open science projects championed by the neuroimaging community critically depends on sustained public investment in methods development, maintenance, and dissemination.

This work aligns with a growing trend in neuroimaging that leverages unprecedented computational resources and vast datasets to revisit foundational assumptions that underlie modern research. While the conceptual core of this work may seem simple, to our knowledge it represents the first comprehensive effort to catalog a wide range of typical fMRI effect types with as much precision as is currently obtainable and necessitated the development of new statistical theory for effect size estimation. We expect that this is just a start; this study lays the groundwork for future expansions that incorporate more diverse datasets, as well as other empirically informed tools for fMRI. By grounding study planning in evidence-based benchmarks for neuroimaging, this work enables more robust and reproducible research essential for understanding the human brain in health and disease.

## Methods

### Datasets

Seven large-scale datasets (n = 100–40,000; 52,979 total participants) were selected based on availability of functional neuroimaging data and sample sizes >100 participants. Datasets spanned developmental stages from childhood and adolescence (PNC, ABCD, HBN) to young adulthood (SLIM, HCP, HCP-EP) and mid-to-older adulthood (UKB), including both typically developing populations and clinical cohorts. Imaging protocols included resting-state and task-based fMRI with diverse phenotypic assessments (Figure 1; detailed study characteristics in **Supplementary Table 1** and *Supplementary Methods*).

### Preprocessing and subject-level test statistics

#### ABCD, HBN, and PNC:

Structural images were skull-stripped (FSL), and functional images were motion- and slicetime-corrected (SPM8). Subsequent steps occurred in BioImage Suite, including nonlinear registration of structural images to MNI space and linear registration of functional images to structural images. Noise covariates were regressed including linear and quadratic drift, a 24-parameter motion model, mean cerebrospinal fluid (CSF), white matter (WM), and global signal (GS). Data were temporally filtered (Gaussian cutoff ≈ 0.12Hz), and functional connectivity was estimated as Fisher z-transformed Pearson correlations between mean timecourses for Shen region pairs.

#### HCP connectivity:

Minimally preprocessed data were obtained from the HCP repository, which included gradient correction, motion correction, fieldmap correction, and linear registration. Subsequent processing in BioImage Suite included nuisance regression (linear/quadratic drift, 24-parameter motion model, CSF/WM/GS), temporal filtering (Gaussian cutoff ≈ 0.12Hz), and nonlinear registration to MNI. Functional connectivity was calculated as above, with right-left and left-right phase encoding connectomes calculated separately and averaged.

#### HCP activation:

Beta coefficient maps were obtained from the repository. Volume-based preprocessing included minimal preprocessing, MNI nonlinear registration (FNIRT), intensity normalization, and smoothing (4mm FWHM). Activations were estimated via general linear modeling with task predictors (canonical HRF) and temporal derivatives as confounds, highpass filtering (200s cutoff), and prewhitening.

#### SLIM:

Connectivity matrices were obtained from the repository. Processing included discarding first 10 volumes, slice-timing correction, motion correction, normalization, resampling to 3mm, smoothing (6mm FWHM), linear detrending, bandpass filtering (0.01-0.08Hz), and nuisance regression (six motion parameters, mean CSF/WM/GS) using DPARSF.

#### UKB:

Connectivity matrices were obtained from the repository. Processing included MNI152 warping (FNIRT), tissue segmentation (FAST), motion correction (MCFLIRT), intensity normalization, highpass filtering (σ=50s), and artifact removal (ICA-FIX). Group-ICA (dimensionality 25) produced network nodes for dual-regression based connectivity, calculating correlations between node timecourses.

### Group-level test statistics

Subject-level maps were analyzed using three statistical tests: one-sample t-test, two-sample t-test, and correlation. Three motion adjustment strategies were evaluated: no correction, statistical control (inclusion of motion as a predictor), and thresholding (exclusion of subjects with > 0.1 mm mean frame-to-frame displacement, previously shown to exclude the top 5% of high motion subjects in the HCP; unpublished). Three scales of inference were examined: edge-/voxel-level (mass univariate), network-level (pooled within Shen 10-network atlas), and multivariate whole-brain level using Canonical Correlation Analysis or Hotelling's T^2^ test. Analyses resulted in mass univariate t-statistics or correlation coefficients.

### Effect size conversion

Test statistics were converted to Cohen's *d* following standard conventions^14^. For the one-sample t-test, $d_{s}=\frac{t}{\sqrt{n}}$ and $SE_{d}=\sqrt{\frac{1}{n}+\frac{d^{2}}{2n}}$. For the two-sample t-test, $d_{s}=t\sqrt{\left(\frac{1}{n_{1}}+\frac{1}{n_{2}}\right)}$ and $SE_{d}=\sqrt{\frac{n_{1}+n_{2}}{n_{1}\cdot n_{2}}+\frac{d^{2}}{2(n_{1}+n_{2})}}$. For correlation, $d_{s}=\frac{2r}{\sqrt{\left(1-r^{2}\right)}}$ (cf. ^14^) and $SE_{d}=\sqrt{\frac{n_{1}+n_{2}}{n_{1}\cdot n_{2}}+\frac{d^{2}}{2(n_{1}+n_{2})}}$, taking group sizes to be equal (i.e, $n_{1}=n_{2}=n∕2$). Multivariate effects were also converted to d following the above conversions.

### Meta-analysis of spatial maps

Mass univariate meta-analyses of Cohen's d used multilevel random-effects models (metafor package) at each brain location within outcome categories. The model $y_{ij}=\mu +u_{i}+v_{ij}+\epsilon_{ij}$ included random effects for dataset ($u_{i}$) and study within dataset ($v_{\text{ij}}$), with known sampling variances $V_{\text{ij}}={{\text{SE}}^{2}}_{\text{ij}}$. Parameters were estimated using restricted maximum likelihood estimation with multiple optimizers applied sequentially if convergence failed.

### Estimating cross-brain effect size distributions

To correct for magnitude inflation of the cross-brain effect size distribution due to sampling error, we modeled how observed effect size variability changes with sample size. We formulated a relationship between the observed (uncorrected, mass univariate) cross-brain variance $var({\hat{\theta }}_{i})$ and true cross-brain variance ${\tau_{\eta }}^{2}$ as

$$var({\hat{\theta }}_{i})={\tau_{\eta }}^{2}+k^{2}\phi^{2}∕n$$

where $\phi^{2}$ is the population variance, k is the number of groups for the test, and n is the total sample size for the study. Within each outcome category, we estimated ${\tau_{\eta }}^{2}$ and $\phi^{2}$ as the coefficients of

$$var({\hat{\theta }}_{i})∼1+\frac{k^{2}}{n}$$

using meta-regression with random intercepts for dataset / outcome category. For zero-centered normal distributions, the expected magnitude is given by the CDF for the half normal $E[|\theta_{i}|]=\tau_{\eta }\sqrt{\frac{2}{\pi }}$, with 68% of effects falling below $\tau_{\eta }$ in magnitude.

### Power for a distribution of effect sizes with multiple testing correction

Given a cross-brain distribution of effect sizes, the expected number of effects surpassing a target power threshold is derived as

$$\;1\text{-sample},2\text{-sided:}\;P(\delta >\delta^{*})=\tilde{\Phi }\left(\frac{\left({\tilde{\Phi }}^{-1}(p^{\prime })-\Phi^{-1}(\beta )\right)-\sqrt{\frac{n_{total}}{4}}\mu_{d}}{\sqrt{\frac{n_{total}}{4}}\sigma_{d}}\right)\;2\text{-sample},2\text{-sided:}\;P(\delta >\delta^{*})=2\tilde{\Phi }\left(\frac{\left({\tilde{\Phi }}^{-1}(p^{\prime }∕2)-\Phi^{-1}(\beta )\right)-{\left(\frac{1}{n_{1}}+\frac{1}{n_{2}}\right)}^{-\frac{1}{2}}\mu_{d}}{{\left(\frac{1}{n_{1}}+\frac{1}{n_{2}}\right)}^{-\frac{1}{2}}\sigma_{d}}\right)$$

where $n_{total}$ is the total sample size, $n_{1}$ and $n_{2}$ are the sample sizes for each group, $p^{\prime }$ is the p-value threshold for rejecting the null, β is the target power level, $\tilde{\Phi }(t)$ is the standard normal complementary CDF, and effect sizes are normally distributed with mean $\mu_{d}$ and variance $\sigma_{d}^{2}$. For an uncorrected test we use $p^{\prime }=0.05$ and for Bonferroni correction $p^{\prime }=0.05∕m$, given m variables. For false discovery rate (FDR) control, $p^{\prime }$ depends on the data; thus we derive the following expression for the FDR power implicit function at level $\alpha_{FDR}$ for a 2-sided test as $\pi_{0}p^{\prime }+\pi_{1}F_{1}(p^{\prime })=\frac{p^{\prime }}{\alpha_{FDR}∕2}$, and the alternate p-value distribution ($F_{1}$) for 1- and 2-sample tests as

$$\;1\text{-sample},2\text{-sided:}\;F_{1}(t)=2\tilde{\Phi }\left(\frac{{\tilde{\Phi }}^{-1}(t)-\mu_{d}\sqrt{n}}{\sqrt{1+n\sigma_{d}^{2}}}\right)\;2\text{-sample},2\text{-sided:}\;F_{1}(t)=2\tilde{\Phi }\left(\frac{{\tilde{\Phi }}^{-1}(t)-{\left(\frac{1}{n_{1}}+\frac{1}{n_{2}}\right)}^{-\frac{1}{2}}\mu_{d}}{\sqrt{1+{\left(\frac{1}{n_{1}}+\frac{1}{n_{2}}\right)}^{-\frac{1}{2}}\sigma_{d}^{2}}}\right).$$

Full derivations available in *Supplementary Methods*.

## Supplementary Material

This is a list of supplementary files associated with this preprint. Click to download.

- SIMethods.pdf
- SITablesXFigures.docx

## Data Availability

All raw data used in the present study have been made publicly available by the contributing repositories in accordance with data use and access regulations set by the respective consortia, summarized as follows. The Northeastern University and Yale University Human Research Protection Programs approved secondary analyses of these datasets. Human Connectome Project (HCP) and Human Connectome Project Early Psychosis (HCP-EP) data are available through the HCP repository (https://www.humanconnectome.org/study/hcp-young-adult ; https://www.humanconnectome.org/study/hcp-early-psychosis). Users must agree to data use terms before accessing ConnectomeDB; details are provided at https://www.humanconnectome.org/study/hcp-young-adult/data-use-terms. UK Biobank data are available to approved researchers conducting health-related research in the public interest through https://www.ukbiobank.ac.uk/. This research was conducted using the UK Biobank Resource under application number 140089. Adolescent Brain and Cognitive Development (ABCD) data are available through the NIH Brain Development Cohorts (NBDC) Data Hub (https://data.NBDC.nih.gov/). The ABCD data used in this report came from Release 2.0.1, NDA Study DOI: 10.15154/1504041. Philadelphia Neurodevelopmental Cohort (PNC) data are available through dbGaP under accession phs000607.v1.p1 (https://www.ncbi.nlm.nih.gov/projects/gap/cgi-bin/study.cgi?study_id=phs000607.v1.p1) for authorized researchers with approved Data Use Certification. Healthy Brain Network (HBN) data are available through the Child Mind Institute LORIS database (http://fcon_1000.projects.nitrc.org/indi/cmi_healthy_brain_network/) for approved researchers. Southwest University Longitudinal Imaging Multimodal (SLIM) data are available under Creative Commons Attribution-NonCommercial license through the International Data-sharing Initiative (https://fcon_1000.projects.nitrc.org/indi/retro/southwestuni_qiu_index.html).

## Code availability

All code used for running statistical analyses has been made publicly available on GitHub (https://github.com/neuroprismlab/calculate_effeX ; https://github.com/neuroprismlab/BrainEffeX_utils ; https://github.com/neuroprismlab/crossbrain_effects ). Initial group-level statistics were obtained with Matlab, and all subsequent statistical estimation was conducted in R. Note that the present study relies on both Matlab and R, since Matlab is often preferred by the neuroimaging community for preparing data for contribution and fast matrix manipulation, whereas R is preferred for its statistical infrastructure and its popular Shiny web app building framework. The BrainEffex interactive R Shiny app (https://neuroprismlab.shinyapps.io/BrainEffeX/) has been developed to facilitate more detailed visualization, exploration, and downloading of the study-specific effect size maps, meta-analyses, and metadata (DOI: 10.5281/zenodo.16882652; Shearer et al., 2025). The BioImage Suite command line software used for processing is freely available at (https://bioimagesuiteweb.github.io/webapp/index.html).
